# Color Blindness

**Published:** 1871-01

**Authors:** 


### COLOR BLINDNESS.

This is an awkward and exceedingly embarrassing imperfection of vision. Very many persons are "color blind" without being aware of it. Physicians who are in the habit of examining the eyes of a great number of people yearly, discover that about one person in every twenty, is color blind—that is, unable to distinguish one or more colors, or mistakes one for another. We find that one in every fifty-five persons is unable to distinguish red from green. It will at once be perceived that such persons are wholly unfit for duty as Rail Road operatives, or for any position where color signals are employed. Doubtless, very many accidents occur from the inability of Engineers and Pilots to distinguish a red from a green light. For this reason, it occurs to us, that Superintendents of Rail Roads or Steamboats, should have their Engineers, Conductors and Pilots thoroughly examined with reference to their being able to distinguish colors; for as we before remarked, many are afflicted with color blindness without being aware of it.

We have frequently seen Accountants who were unable to distinguish the red from the blue line in their ledgers, and we once knew an Artist who would persist in painting his skies green and his grass blue, and finally had to abandon his painting and adopt drawing with crayons, and pencil, where only one color was used. The difficulty is much more prevalent than people are aware of, and for this reason tests should always be made of the eyes of persons who are about to accept any position where the reading of colors aright, is imperatively demanded.

ONE page of advertising is unavoidably crowded out this number.

